# Effect of Aluminum Treatment on Proteomes of Radicles of Seeds Derived from Al-Treated Tomato Plants

**DOI:** 10.3390/proteomes2020169

**Published:** 2014-03-28

**Authors:** Ikenna Okekeogbu, Zhujia Ye, Sasikiran Reddy Sangireddy, Hui Li, Sarabjit Bhatti, Dafeng Hui, Suping Zhou, Kevin J. Howe, Tara Fish, Yong Yang, Theodore W. Thannhauser

**Affiliations:** 1 Department of Agricultural and Environmental Sciences, College of Agriculture, Human and Natural Sciences, Tennessee State University, 3500 John A Merritt Blvd, Nashville, TN 37209, USA; E-Mails: iokekeog@my.tnstate.edu (I.O.); zhujiaye117@gmail.com (Z.Y.); sangisasi@gmail.com (S.R.S.); hli@my.tnstate.edu (H.L.); sbhatti@Tnstate.edu (S.B.); dhui@Tnstate.edu (D.H.); 2 Plant, Soil and Nutrition Research Unit, USDA-ARS, Tower Rd, Ithaca, NY 14853, USA; E-Mails: kjh46@cornell.edu (K.J.H.); tlf26@cornell.edu (T.F.); yy44@cornell.edu (Y.Y.)

**Keywords:** iTRAQ, orbitrap mass spectrometry, Al-enriched tomato seeds, seed germination, root proteomics, functional pathways

## Abstract

Aluminum (Al) toxicity is a major constraint to plant growth and crop yield in acid soils. Tomato cultivars are especially susceptible to excessive Al^3+^ accumulated in the root zone. In this study, tomato plants were grown in a hydroponic culture system supplemented with 50 µM AlK(SO_4_)_2_. Seeds harvested from Al-treated plants contained a significantly higher Al content than those grown in the control hydroponic solution. In this study, these Al-enriched tomato seeds (harvested from Al-treated tomato plants) were germinated in 50 µM AlK(SO_4_)_2_ solution in a homopiperazine-1,4-bis(2-ethanesulfonic acid) buffer (pH 4.0), and the control solution which contained the buffer only. Proteomes of radicles were analyzed quantitatively by mass spectrometry employing isobaric tags for relative and absolute quantitation (iTRAQ^®^). The proteins identified were assigned to molecular functional groups and cellular metabolic pathways using MapMan. Among the proteins whose abundance levels changed significantly were: a number of transcription factors; proteins regulating gene silencing and programmed cell death; proteins in primary and secondary signaling pathways, including phytohormone signaling and proteins for enhancing tolerance to abiotic and biotic stress. Among the metabolic pathways, enzymes in glycolysis and fermentation and sucrolytic pathways were repressed. Secondary metabolic pathways including the mevalonate pathway and lignin biosynthesis were induced. Biological reactions in mitochondria seem to be induced due to an increase in the abundance level of mitochondrial ribosomes and enzymes in the TCA cycle, electron transport chains and ATP synthesis.

## 1. Introduction

Aluminum is not an essential mineral to plants; Al ions (Al^3+^), when at an excessive level, are very toxic to seed germination in both tolerant and susceptible plants [1,2]. Of all the Al-induced phytotoxic symptoms, disruption of cell division and growth within the root apex has the most significant impact on plant growth and yield [3,4]. Thus, many studies are focused on the physiological and molecular activities in the root tip zone related to reducing Al^3+^ phytotoxicity. Tolerance to Al is achieved by avoidance mechanisms (e.g., through secretion of organic acids to bind Al^3+^ near the vicinity of root tips) [5,6,7], and internal resistance, by remodeling cellular processes [8,9] and through apoplastic and symplastic detoxification of internalized Al [10,11]. 

Seed germination is the process by which an embryo transitions into a complete plant. It begins as the root (radicle) becomes the first embryonic organ to emerge from the seed coat; this is followed by elongation of the hypocotyl and ends in expansion of the cotyledon (s) [12,13,14]. Radicle emergence involves both cell division and cell enlargement. Radicle growth (mainly involving cell enlargement) is sensitive to metal toxicity [15]. A study using *Arabidopsis thaliana* shows that *de novo* protein synthesis from the pool of stored mRNA is essential for the completion of radicle protrusion; however, the process can proceed even in the absence of transcription (*de novo* mRNA synthesis) [16]. Pre-incubation of wheat (*Triticum aestivum*) seedlings with low doses of Al increased tolerance to subsequent exposure to lethal concentrations. The study also concluded that synthesis of proteins is essential for acquiring tolerance to Al because addition of the protein translation inhibitor, cycloheximide, completely abolished the induced tolerance to Al toxicity [17]. Therefore, proteome changes in the primary root can directly affect the development of tolerance and may represent the key to understanding the molecular mechanism involved. 

Tomato (*Solanum lycopersicum*) is among the few species that produce very acidic fruits (pH < 3.0 in ripened tomato fruit), thereby providing an environment capable of shifting the equilibrium from the benign Al^2+^ form to the highly toxic Al^3+^. In this study, tomato plants were grown in a hydroponic system supplemented with Al during the reproductive stages (from flowering until fruit ripening). Tomato seeds produced by these plants were considered to be Al-enriched as they contained a higher Al content than those harvested from plants growing in a solution without added Al. Subsequently, the Al-enriched seeds were germinated in an Al solution, and a proteomics analysis of their radicles was performed to identify proteome changes in response to the Al treatment as a means to identify candidate proteins that could play a key role in acquiring Al tolerance. 

## 2. Experimental

Tomato (*S. lycopersicum* cv. Micro-Tom) plants were grown in a hydroponic culture system. As soon as plants started to set fruits (pea-sized fruits were seen on the first fruit cluster), AlK(SO_4_)_2_ was added up to a final concentration of 7.2 µM of Al^3+^ activity [or 50 µM AlK(SO_4_)_2_]. The pH of the solution was tested daily using pH strips (Fisher Scientific) and the solution was refreshed weekly or when the pH increased to 5.0. Tomato fruits were harvested periodically when the color turned red. To collect tomato seeds, fruits were wrapped in paper towels to squeeze out all the tomato juice. After removal of the gelatinous sack tissues, seeds were soaked in 50% bleach for 5 min followed by three rinses in autoclaved water. Seeds were stored at 4 °C until analysis. These field experiments were performed for two seasons. Mineral analysis of seed tissues (embryo and seed coat separately) found that the Al content of embryo was 10–15 mg per kg dry weight (DW) for seeds derived from Al-treated plants, and it was 6–8 mg per kg DW for those harvested from plants growing in the same hydroponic system but without adding AlK(SO_4_)_2_. In this experiment, it was noticed that control samples including roots, leaves and seeds also contained Al although the content level was much lower than the treated samples. Consistently in the two-season experiments, the Al-treated embryos contained a significantly (*p* < 0.01 using *t*-test) lower amount of boron (B) and iron (Fe) [18]. 

A seed germination assay was conducted to test the effect of Al treatment on improving Al tolerance of the next-generation offspring. Seeds (Al-enriched) that were harvested from Al-treated plants were germinated on wet filter paper soaked in a 50 mM homopiperazine-1,4-bis(2-ethanesulfonic acid) (Homopipes) buffer containing 50 µM AlK(SO4)_2_ (pH 4.5–5.0) in the Al-treated group, as opposed to the buffer only in the control treatment. The germination was carried out at 25 ± 2 °C. The lengths of radicles were measured on the third day of germination. There was no significant difference in terms of radicle length between Al-treated (5 ± 1 mm) and untreated groups (4 ± 1 mm). These results indicate that radicle elongation growth was not affected by the presence of Al in the solution for these seeds. 

For preparation of this study, seeds were germinated under the same conditions, with three biological replicates for treated and control experiments. Each biological replicate consisted of 100 seeds with 10 seeds wrapped in one filter paper sandwich. Radicles protruding from seed coat were dissected using a sharp blade on the third day after germination. Tissues were frozen in liquid nitrogen and ground into a fine powder immediately after harvest. 

### 2.1. Protein Extraction and Isobaric Tags for Relative and Absolute Quantification Labeling

For protein extraction, tissue powder was washed sequentially in 10% TCA/acetone, 80% methanol/0.1 M ammonium acetate, and 80% acetone with centrifugation to pellet the powder after each wash. The protein was then extracted in a phenol (pH 8.0) and dense SDS buffer [30% sucrose, 2% SDS, 0.1 M Tris-HCl, pH 8.0, 5% beta-mercaptoethanol (v/w)]. After incubation at 4 °C for 2 h, the mixture was centrifuged at 16,000 *g* at 4 °C for 20 min. Protein in the upper phenol phase was precipitated in 0.1 M ammonium acetate in methanol after incubation overnight at −20 °C. Protein pellets were washed in methanol and acetone and were then dissolved in a buffer of 500 mM triethylammonium bicarbonate (TEAB) and 2 M urea, and 0.1% SDS and a proteinase inhibitor cocktail for plant tissue (100 × dilution in the extraction buffer) (Sigma, St. Louis, MO, USA). Protein concentration was determined using a Bradford assay kit (Bio-Rad, Hercules, CA, USA). 

One hundred µg of protein from each sample was digested with trypsin and then labeled as previously described [19] following the instructions accompanying the 8-plex iTRAQ^®^ labeling kit (AB SCIEX, Framingham, MA, USA). The treated samples were labeled with tags 113, 114 and 115 and the control samples with 116, 117 and 118 were combined. Unbound tags and SDS were removed through cation exchange cartridge (AB SCIEX), and salts were removed using reverse-phase solid-phase extraction procedure involving 1-cm^3^, 50-mg cartridges following the manufacturer’s instructions (Sep-Pak C_18_; Waters, Milford, MA, USA). Peptides were eluted in 500 μL 50% (v/v) acetonitrile with 0.1% TFA and dried under vacuum. 

These peptide samples were subjected to a first dimension of high pH Ultra Performance Liquid Chromatography (UPLC*)* separation using an Acquity UPLC System (Waters) coupled with a robotic fraction collector (Probot; Dionex, Sunnyvale, CA, USA) [19]. One hundred micrograms of the multiplexed sample was injected and fractionated into 48 fractions in a 96-well plate. The 48 fractions were concatenated to yield 16 samples pools by pooling every 16th sample. These were dried at reduced pressure using a CentiVac Concentrator (LabConco, Kansas City, MO, USA). For the low pH 2nd dimension, low pH reverse-phase (RP) chromatography was employed. Dried samples were reconstituted with 15 µL of 2% acetonitrile with 0.5% formic acid. Nano-LC separations of tryptic peptides were performed as described previously [20,21]. The eluent from the analytical column was delivered to the LTQ-Orbitrap Elite (Thermo-Fisher Scientific, Waltham, MA, USA) via a “Plug and Play” nano ion source (CorSolutions LLC, Ithaca, NY, USA). The mass spectrometer was externally calibrated across the *m/z* range from 375–1,800 with Ultramark 1621 for the FT mass analyzer, and individual runs were internally calibrated with the background polysiloxane ion at *m/z* 445.1200025 as a lock mass. 

The Orbitrap Elite was operated in the positive ion mode with nanosource voltage set at 1.7 kV and source temperature at 250 °C. A parallel DDA mode was used to obtain one MS survey scan with the FT mass analyzer, followed by isolation and fragmentation of the 15–20 most abundant, multiply-charged precursor ions with a threshold ion count higher than 50,000 in both the LTQ mass analyzer and the high energy collisionally induced dissociation (HCD)-based FT mass analyzer at a resolution of 15,000 (fwhm *m/z* 400). MS survey scans were acquired with resolution set at 60,000 across the survey scan range (*m/z* 375–1800). Dynamic exclusion was utilized with repeat count set to 1 with a 40 s repeat duration; exclusion list size was set to 500, 20–30 s exclusion duration, and low and high exclusion mass widths set to 1.5. Fragmentation parameters were set with isolation width at 1.5 *m/z*, normalized collision energy at 37%, activation Q at 0.25. Activation time for HCD analysis was 0.1 min. All data were acquired using XCalibur 2.1 (Thermo-Fisher Scientific).

### 2.2. Data Processing, Database Searching and iTRAQ Quantitation

Proteome Discoverer v 1.4 was used to convert raw spectral data files for each iTRAQ experiment into a merged peak list (mgf format) containing all 2nd dimension fractions for each tomato experiment for subsequent database searching. Mascot Daemon v. 2.3.2 was used to query .mgf files against an iTAG 2.3 tomato protein database [22]. Trypsin was selected as the enzyme with 1 missed cleavage allowed. Methylthiolation of cysteine, oxidation of methionine, and deamidation of asparagine and glutamine were set as variable modifications. Peptide charge was set to 2^+^, 3^+^, and 4^+^. Precursor tolerance was set to 10 ppm, while fragment tolerance was set to 100 mmu. The instrument selected was ESI-FTICR. The iTRAQ quantitation method utilized a weighted protein ratio type, featured outlier removal, and required a minimum of 2 peptides for protein quantitation. Summed normalization was used. For the iTRAQ 8-plex labeling, N-terminal and lysine modification with iTRAQ were set as fixed modifications, and tyrosine labeling was set as a variable modification. Upon completion of searching, each report was opened and results were exported after setting the ion score filter to 0.1, thereby exporting only results with an expectation value below 0.1, specifying unique peptides only. Only the highest scoring matches to a particular peptide sequence, listed under the highest scoring protein containing that match, were considered. 

### 2.3. Protein Quantification, Statistics, and Protein Functional Analysis

For a protein to be included in the quantitative analysis, it was required that at least two unique peptides (with the normalized intensity levels raw intensity >20) were identified in all the six biological samples (three biological replicates each in Al-treated and control groups). The normalized peak intensities of reporter ions of constituent peptides were log_2_ transformed. Then, log_2_ fold values from all constituent peptides were subjected to *t*-test (general linear model procedure) followed by false discovery rate (FDR) corrections to test the statistical significance of the difference in normalized abundance of each protein between Al-treated and control sample groups [19]. The log_2_ transformed abundance ratios were then fit to a normal distribution. Two standard deviations (at a 95% confidence level) of the log_2_ fold (from treated to untreated control) in protein abundance were used as the cutoff threshold for significantly changed proteins. Statistical analyses were performed using SAS (version 9.3; SAS Institute, Cary, NC, USA).

MapMan [23] was used to associate the identified tomato proteins with cellular process and metabolic pathways using iTAG 2.3 tomato protein database downloaded from the MapMan website. The putative functions of the identified proteins were also discussed based on relevant information from literature and database searches on tomatoes and other plant species.

## 3. Results and Discussion

**Figure 1:**
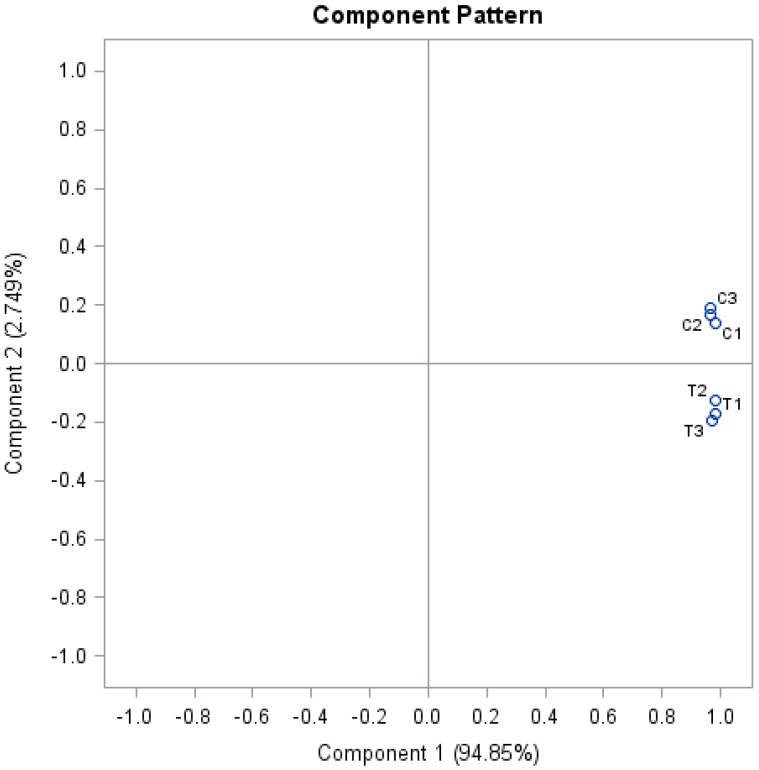
Principal component distribution (PCA) of proteomes from Al-treated tomato radicle. (Tryptic peptides from six biological samples were labeled with iTRAQ tags (treated samples with tags 113, 114 and 115 and the control samples with 116, 117 and 118). The intensity of reporter ions of peptides from mass spectrometry analysis was log-transformed (base, 2). Protein samples were clustered based on the distribution of log_2_ fold change values of all peptides in the six tagged samples. Three control biological replicates: C1, C2, C3; three treated biological replicates: T1, T2, T3).

**Figure 2:**
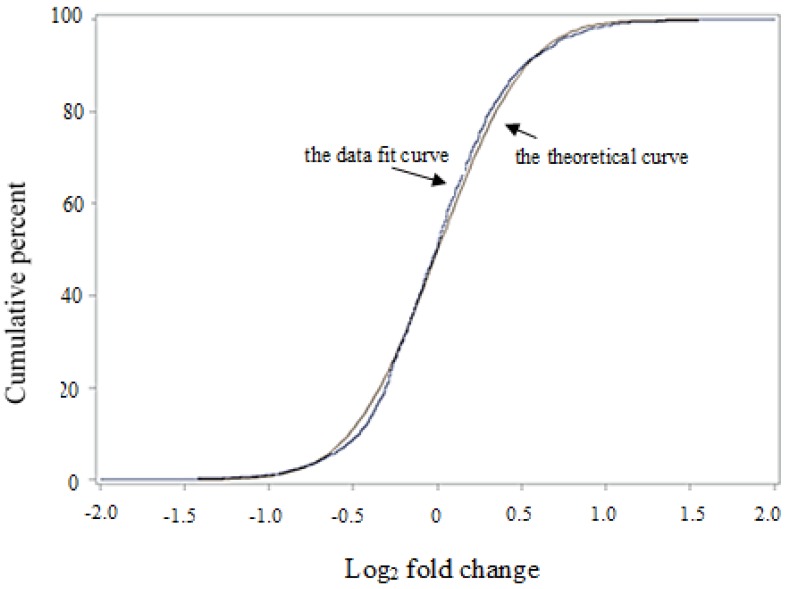
The normal distribution fit of the log_2_ fold values of proteins from Al-treated tomato radicles. (Tomato seeds were germinated in 50 mM Homopipes (pH 4.5) buffer supplemented with 50 µM AlK(SO_4_)_2_ and the control solution contained the buffer only. Tryptic peptides were labeled with iTRAQ tags (treated samples with tags 113, 114 and 115 and the control samples with 116, 117 and 118) followed by analysis using mass spectrometry. The reporter ion intensity of all the tags was log-transformed and the log_2_ fold changes of protein from Al-treated and untreated tomato samples were plotted against a theoretical normal distribution in SAS program. The purple-colored is the theoretical curve and the blue-colored is the data fit curve).

In this study, 3,160 proteins, meeting the quantification analysis criteria of two or more peptides, were identified in all six biological samples. The spectral intensity of each peptide was transformed into log (base 2) values, and principal component analysis (PCA) (Figure 1) separated the treated and control triplicate groups, which indicates that there is a systematic difference in protein composition between the two groups. The low percentage in component 2 compared to component 1 (2.7% *vs*. 94.87%) indicates that differences among proteins (or peptides of the same protein) are much greater which is understandable as the proteome is comprised of proteins of different abundance (high *versus* low content levels). Data of log_2_ fold change of proteins from treated to untreated groups fit into a near normal distribution (Figure 2). After *t*-test and FDA corrections, 139 proteins were found to be significantly changed from untreated to treated root samples (*p* ≤ 0.05) and the fold change passed the threshold of a two standard deviation (>±0.82). Fifty-two proteins were repressed and 87 proteins were induced, and Al-induced changes in protein abundance were given as the ratio between treated and non-treated control groups which is the antilogarithm of log_2_ (fold) (Appendix Table A1). 

### 3.1. Al Treatment-Induced Proteome Changes and the Associated Cellular and Molecular Functions

**Figure 3:**
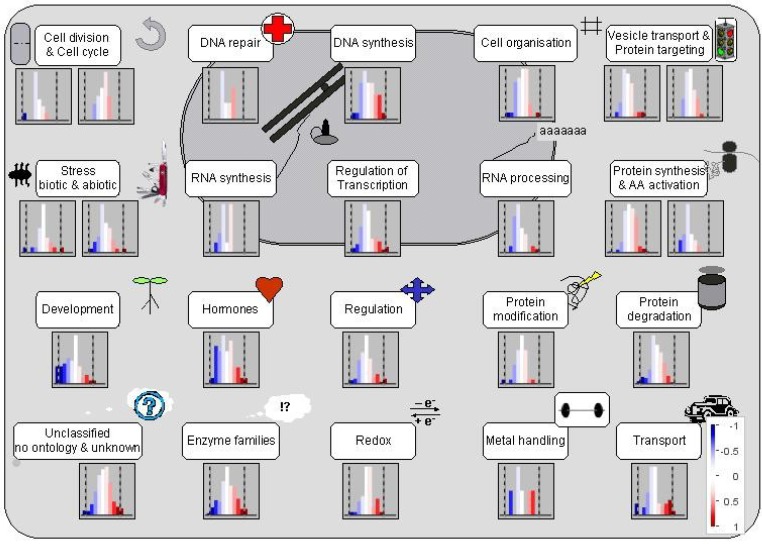
Cell function overview of proteins from radicles of Al-treated tomato seeds. (The graphic was generated using the Cell-Function Overview and Slyc-iTAG2.3 as the reference database in MapMan. The intensity of the color change corresponds to the scale created based on log_2_ fold of protein from Al-treated to untreated groups.)

Using MapMan, tomato root proteins were clustered into 20 cellular functional pathways (Figure 3). In each of the functional groups, there were proteins repressed, induced and unchanged (the intensity of the color change corresponded to the log_2_ fold change of respective proteins from treated to untreated groups). The majority of the 20 functional pathways contained significantly changed proteins. These results are in agreement with previous findings that many genes (and gene products) located in multiple genome regions and participating in various cellular activities could be involved in the modulation of plant responses to Al stress [24,25,26,27]. 

For those significantly changed proteins, additional manual searches of literature and other plant databases were performed to identify their putative roles in Al and secondary cellular stress. These proteins were divided into eight groups by combining MapMan classification (based on known protein functions) and putative functions derived from other sources. 

#### 3.1.1. Mobilization of Seed Storage Proteins in Al-Treated Tomato Radicles

Mature seeds contain many species of hydrophilic proteins, such as dehydrins, globulins, and late embryogenesis abundant proteins (LEA). During seed germination, these seed proteins play a key role in maintaining intracellular water balance by controlling water uptake, and they are also recycled to provide a nitrogen source for the germinating embryo. In this study, Al-treated radicles were found to contain a lower level of hydrophilic proteins, such as globulin, vicilin, LEA, seed biotin-containing protein SBP65, dehydrin, and small hydrophilic plant seed protein. These results suggest that the Al treatment induced more active catabolism of hydrophilic proteins or inhibited transport of those proteins from cotyledons to the growing radicles. Both processes can result in a low hydrophilic protein content in the Al-treated radicles. 

In addition, two seed oil body-associated proteins, caleosin and oleosin, were also repressed in Al-treated radicles. These lipid body-binding proteins play a key role in the degradation of storage lipid during seed germination [28,29]. A decrease in oleosin content can lead to coalescence of lipid bodies which is harmful to cells, or it may make the radicle more susceptible to dehydration as these proteins also affect tissue tolerance to desiccation [30]. In summary, changes in proteins of this group will make tomato more sensitive to dehydration stress, which can happen under excessive salt and drought stresses. This could be one of the major molecular mechanisms by which Al toxicity can exacerbate the impact of other environmental stress factors during seed germination. 

#### 3.1.2. Proteins Involved in Cell Organization, Cell Division and Cell Cycle

In the Al-treated radicles, proteins affecting cell division cycle (protease ftsH homolog) and cell skeleton structure (actin and tubulin) were repressed. On the other hand, proteins in programmed cell death (PCD) or related processes were induced. These are the RPM1 interacting proteins which are essential for hypertensive cell death in reaction to pathogens [31] and the vesicle-associated membrane family protein which has a critical role in regulating execution of PCD by affecting the rate of membrane recycling, especially under oxidative stress [32]. 

#### 3.1.3. Proteins Involved in Regulation of Transcription

Expression of genetic materials provides the basis for all physiological traits. One of the first critical steps is the regeneration of mature mRNA (gene transcripts). In tomato radicles, a number of transcription factors were affected by Al treatment. Several C2H2 zinc finger family proteins were induced, but CCHC zinc finger, CCCH-type zinc finger and ZF-HD class zinc finger-homeodomain proteins were repressed. Additional induced proteins are Myb transcription factor, BolA-like protein and bZIP transcription factor. DNA silencing and mRNA decay are both important mechanisms in regulating gene expression, especially under stress conditions. Proteins in these categories were induced in the Al-treated tomato radicles, and include DNA (cytosine-5-)-methyltransferase 3, U6 RNA-associated Sm-like proteins and LSm6. Two proteins in the nonsense-mediated mRNA decay (NMD) pathway were also identified; they are eukaryotic translation initiation factor SUI1, and eukaryotic translation initiation factor 4 gamma-MIF4-like. Activation of these mechanisms can help plant cells to rid themselves of aberrant proteins and transcripts, and ensure a “healthy mRNA pool” in the Al-treated tissues. 

#### 3.1.4. Proteins Affecting Protein Synthesis and Post-Translational Modification

Plants have three separate sets of genomes in chloroplasts, mitochondria, and cell nucleus. While nuclear genes are translated in the cytoplasm, the mitochondria and chloroplasts each contain its own translation machinery within the respective organelles [33,34]. Changes in ribosomal proteins suggest that protein translation in plastids and mitochondria may be more active as ribosomal proteins annotated to those organelles were induced, which include 30S ribosomal protein S7, ribosomal protein L24 and L27 for chloroplasts, and mitochondrial ribosomal protein L37 and L15 and peptidyl-tRNA hydrolase ICT1. In mitochondria, all the proteins in the electron transport chain and ATP synthase are synthesized by mitochondrial ribosomes [35]. The Al-induced ribosomal protein expression could have some effect on mitochondrial functions (which will be discussed later). 

Cytoplasmic ribosomes which are responsible for the translation of nuclear-genome encoded genes revealed more complex changes. Some proteins were induced including ribosomal protein S21e and L32e, whereas others were repressed including ribosomal protein S26e, L15e and L19/L19e. Differential relative abundances of these ribosomal proteins were also found in tomatoes under other abiotic stresses [19,36]. 

A large number of proteins undergo post-translational modification to generate biologically active forms and/or to be targeted into correct subcellular organelles. One major post-translation modification is protein phosphorylation which is catalyzed by kinase and the dephosphorylation catalyzed by phosphatase. In the Al-treated tomato radicles, a serine/threonine kinase protein was induced, whereas serine/threonine-specific protein phosphatase was repressed. Protein serine/threonine phosphatases are implicated in the regulation of apoptotic pathways [37,38]. A study on wheat showed that the active function of protein kinase seems to be essential in alleviating Al-induced root inhibition as protein phosphorylation was found to be involved in the Al-responsive malate efflux in root tip [39]. Therefore, changes in this pair of enzymes may have some effect on different aspects of cellular processes under Al stress. 

#### 3.1.5. Proteins Involved in Protein Degradation and Modification

The ubiquitin-proteasomal degradation pathway is essential for many cellular processes, including the cell cycle, the regulation of gene expression, and responses to oxidative stress. The proteasome complex consists of ubiquitin-activating enzyme (E1), ubiquitin-conjugating enzyme (E2) and ubiquitin ligases (E3). Among the three subunits, E3 controls the specificity of protein degradation. While no significant changes were found in E1, E2, or E3 in the Al-treated tomato radicles, the COP9 signalosome (CSN) subunit 6 was induced. CSN is the protein regulating the function of ubiquitin E3; it is rapidly emerging as a key player in the DNA-damage response, cell-cycle control and gene expression, and plant response to environmental stimuli and stresses [40,41,42]. This is the first time that this protein has been found to be regulated by Al stress.

#### 3.1.6. Proteins Involved in Hormone Metabolism and Signaling

In the Al-treated radicles, proteins involved in the biosynthesis of three hormones and their signaling pathways were induced. This includes ethylene (1-aminocyclopropane-1-carboxylate oxidase and multiprotein bridging factor 1) and abscisic acid (ABA) signaling pathway (ABA/WDS-induced protein). The jasmonate pathway enzymes were repressed, which include lipoxygenase and allene oxide synthase. Proteins for the biosynthesis of gibberellin were repressed (gibberellin 3 beta-hydroxylase 2–3 and gibberellin 2-oxidase 2), but several gibberellin-regulated proteins were repressed or induced (0.59–2.06-fold). 

In general, ABA-related genes are more highly expressed when germination is inhibited and the hormone (ABA) inhibits radicle emergence [43,44]. In contrast, GA-related genes are activated during seed germination [43]. In this study, tomato seeds germinated at the same rate under Al-treated and non-treated conditions, therefore, there was no correlation between this physiological process and changes in these ABA- and GA-related proteins. These results indicate that a more complex hormone signaling and interaction mechanism is involved in radicle growth during tomato seed germination.

#### 3.1.7. Proteins in Signal Transduction

In the Al-treated tomato radicles, mitogen-activated protein kinase (MAPK), a key enzyme in MAPK signaling pathway, was strongly induced. This enzyme plays a key role to communicate an external signal from a receptor on the surface of the cell to the DNA in the nucleus of the cell [45]. Earlier studies using cell suspension cultures of coffee (*Coffea arabica*) found that the MAPK was activated by the oxidative burst induced by Al treatment but this protein is not necessarily associated with Al tolerance [46,47]. Additionally, several calcium-binding proteins that participate in the secondary calcium cell signaling pathways to mediate intracellular stress responses were also induced in the Al-treated tomato radicles. Therefore, the higher abundance of MAPK and Ca-binding proteins in Al-treated tomato radicles may also have a role in enhancing plant tolerance to the secondary cellular stresses induced by Al stress. 

More importantly, the rapid alkalinization factor (RALF) proteins were induced in the Al-treated tomato radicles. RALF is a 5-kDa ubiquitous polypeptide initially isolated from tobacco leaves that induces a rapid alkalinization of the culture medium of tobacco suspension-cultured cells and a concomitant activation of an intracellular mitogen-activated protein kinase [48]. A synthetic tomato RALF homolog peptide, when supplied to germinating tomato and *Arabidopsis thaliana* seeds, caused an arrest of root growth and development [49]. This is the first finding that Al induced an increase in the endogenous level of RALF in tomato radicles, which might also have some roles in regulating cell cycle under the stress condition.

#### 3.1.8. Stress Proteins

The induced proteins include the universal stress protein family, major latex-like proteins and germin-like protein and wound/stress protein. Some of these proteins were also induced during tomato seed germination of normal seeds (seeds harvested from non-treated plants) in Al-treated solution [27]. However, it seems that Al-treated radicles contained a lower level of several heat shock proteins, including Hsp40, DnaJ, DnaJ 2, Hsp 70, ClpB chaperone, class IV HSP and class I HSP, as well as a low-temperature-induced 65 kDa protein. These important protection proteins are mostly induced by stress factors, and they may have a different role under Al stress. 

Oxidative burst is an important secondary cellular stress induced by Al [50]. Among the antioxidant enzymes, thioredoxin and superoxide dismutase, and germin (oxalate oxidase) were induced in Al-treated radicles. In contrast, germin protein was reduced in tomato “Money Maker” treated with the same type of stress [27]. Previous studies indicate that the Al-induced up-regulation of oxalate oxidase gene in the root tip of wheat helps roots to get rid of Al-damaged cells and maintain a healthy epidermal layer of roots, thus protecting the deeper layer of the meristematic and elongation zone that are essential for root growth [51]. Therefore, the induction of germin and germin-like proteins may enhance Al tolerance which was acquired during exposure to Al during seed germination. 

#### 3.1.9. Enzymes in Cellular Metabolism

**Figure 4:**
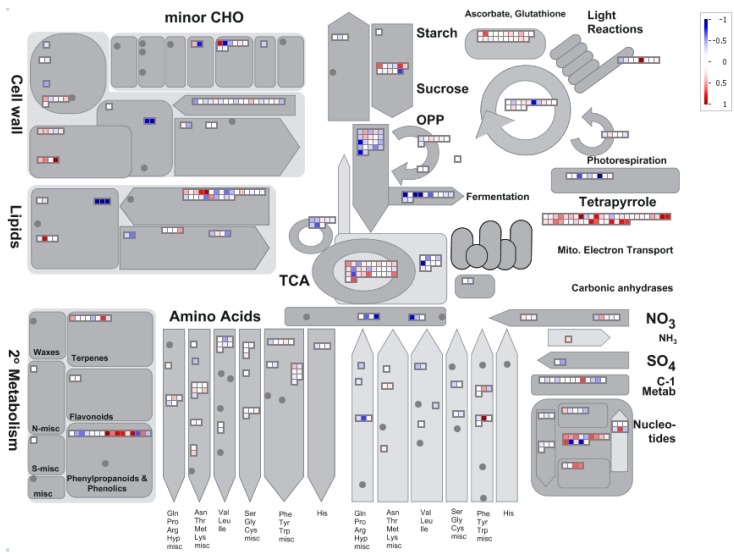
Overview of metabolic pathways in tomato radicles. (The graph was generated using the Metabolism Overview in MapMan [23]. The intensity of the color change corresponds to the scale created based on log_2_ fold of respective protein from Al-treated to untreated tomato radicle tissues.)

Protein changes in different metabolic pathways are shown in Figure 4. In the Al-treated tomato radicles, fermentation and glycolysis pathways were repressed, due to the reduced abundance in pyruvate decarboxylase-2, alcohol dehydrogenase, and an additional 10 enyzmes in glycolysis. Phosphate dikinase in glucogenesis pathway was also repressed. 

The only pathway that was enhanced in Al-treated radicles is the TCA cycle where malate dehydrogenases were induced. The most systematic changes were found in the mitochondrial electron transfer chain (ETC). ETC consists of complex I, II, III, IV, and ATPases for generation of ATP. The induced proteins are localized in NADH-DH.complex I (NADH ubiquinone dehydrogenase), complex II (succinate dehydrogenase assembly factor 2), complex III (biquinol-cytochrome C reductase complex proteins); a class IA/ IB cytochrome to transfer electrons from complex III to complex IV; complex IV (cytochrome c oxidases), and five ATPase proteins (F0 complex subunit D, F1 complex, OSCP/delta subunit, epsilon subunit and delta/epsilon subunit). Such changes suggest that metabolic pathways from sucrose degradation to glycolysis and fermentation could be repressed, but TCA and ETC in mitochondria were induced in tomato radicles under Al treatment. 

Enzymes in glycolysis and fermentation pathways are all encoded by nuclear genes and translated in cytoplasm. As described above, Al induced the repression of several cytosolic ribosomal proteins, which could have affected translation of these proteins. Mitochondial ETC proteins are encoded by mitochondrial genes, and translated within the organelle. Aluminum induced expression of several mitochondrial ribosomal proteins, which may have promoted translation of these proteins in the Al-treated radicles. 

Proteins induced in secondary metabolism include isopentenyl-diphosphate delta-isomerase in the mevalonate pathway (MVA), several *O*-methyltransferases, cinnamyl alcohol dehydrogenase-like protein and caffeoyl-CoA O-methyltransferase in the phenylpropanoids and lignin biosynthesis. These pathways are activated by various biotic and abiotic stresses [19,52,53]. In a previous study of Al treatment of tomato roots, none of these proteins were identified [27]. The use of different analytical methods (2D-DIGE in the previous analysis and iTRAQ in the present one) and variation in the seed proteomes (regular seeds compared to Al-enriched from two tomato cultivars) may be factor(s) causing the identification of different proteins in the two experiments. 

## 4. Conclusions

When subjected to excessive Al and other toxic metals, plants have to cope with the direct ion toxicity and the induced secondary cellular stresses, such as accumulation of reactive oxygen species [50,54] and toxic metabolic aldehydes [55] among harmful biomolecules. Correspondingly, multiple and various cellular pathways are affected during the process [5,56], with concomitant alteration in expression of proteins in multiple functional groups. As shown in this proteomics study, proteins in nearly all of the 20 functional categories displayed significant changes in abundance in the Al treated condition. The Al-induced changes in the proteomes of radicles generated from Al-enriched tomato seeds can be summarized as follows:

1. The Al-treated radicles contained lower abundance of hydrophilic (seed) proteins and oil body membrane proteins, which could reduce the tolerance to dehydration. This could cause the tomato seedlings to be more susceptible to drought and salt and other factors;
2. Mitochondria function was enhanced because of the active protein translational machinery (induction in ribosomal proteins) and TCA–oxidative phosphorylation cycle;
3. The identified proteins include regulatory proteins for gene expression, signaling pathways, cell cycle and programmed cell death.

Aluminum (Al) is ubiquitous in soil being the most abundant metal in the earth’s crust (>8% by weight). When solubilized at pH values below 5.0, it is highly toxic to plants as Al^3+^. Approximately 50% of the world’s potentially arable lands are acidic [57]. A large proportion of the acid soils occur in developing countries in the tropics and subtropics and it has been estimated that the humid tropics account for 60% of the acid soils in the world. Thus, acid soils limit the growth of crops in many developing countries where food production is critical. Acid soils also have a significant impact on U.S. agriculture as approximately 135 million hectares of land in the U.S. are highly acidic. Furthermore, intensive agricultural practices used in the U.S. and in other developed countries, including the widespread use of N fertilization with anhydrous ammonia, can cause significant acidification of surface soils [58], thereby exacerbating an already thorny problem. Thus, there is a genuine need to better understand Al tolerance mechanisms and the genes/proteins that define them, to sustain and enhance crop production on acid soils. The knowledge gained from this and similar studies will provide the scientific underpinning for novel strategies to overcome the challenge of Al stress, and to sustain agricultural production on acid soils. 

## Appendix

**Appendix Table A1:** Induction of significantly changed proteins in radicles of seeds derived from aluminum-treated tomato plants ^z^.

Protein accessions ^y^	Protein name ^x^	log2 fold ^w^ (Treated/control)	Ratio ^v^ Treated/control)
Solyc03g019820.2.1	Aquaporin	−1.88	0.27
Solyc06g034040.1.1	Oleosin	−1.73	0.30
Solyc06g072130.2.1	Aquaporin	−1.64	0.32
Solyc02g086490.2.1	Oleosin	−1.61	0.33
Solyc02g084840.2.1	Dehydrin DHN1	−1.53	0.35
Solyc03g112440.1.1	Oleosin	−1.52	0.35
Solyc06g072670.2.1	Short-chain dehydrogenase/reductase SDR	−1.47	0.36
Solyc06g053740.2.1	Ubiquitin carboxyl-terminal hydrolase	−1.42	0.37
Solyc01g109920.2.1	Dehydrin	−1.35	0.39
Solyc06g065050.1.1	Transmembrane protein 205	−1.33	0.40
Solyc02g077240.2.1	Pyruvate decarboxylase	−1.30	0.41
Solyc12g010920.1.1	Oleosin	−1.28	0.41
Solyc09g082330.1.1	7S vicilin	−1.24	0.42
Solyc12g096930.1.1	Caleosin	−1.18	0.44
Solyc10g008040.2.1	Seed biotin-containing protein SBP65	−1.17	0.44
Solyc11g067250.1.1	Poly (AHRD V1 ***- B9SCR8_RICCO)	−1.11	0.46
Solyc02g085590.2.1	Vicilin	−1.10	0.47
Solyc11g072380.1.1	Vicilin-like protein	−1.09	0.47
Solyc06g075270.2.1	Convicilin	−1.07	0.48
Solyc01g100390.2.1	Pyrophosphate-energized proton pump	−1.06	0.48
Solyc06g009210.2.1	Ribosomal protein L19	−1.03	0.49
Solyc09g025210.2.1	Legumin 11S-globulin	−1.03	0.49
Solyc05g053140.2.1	26S proteasome non-ATPase regulatory subunit 13	−1.02	0.49
Solyc10g076510.1.1	Pyruvate decarboxylase	−1.01	0.50
Solyc05g053120.1.1	Glucosyltransferase	−0.99	0.50
Solyc08g014000.2.1	Lipoxygenase	−0.98	0.51
Solyc08g078850.2.1	L-lactate dehydrogenase	−0.95	0.52
Solyc01g009660.1.1	Low-temperature-induced 65 kDa protein	−0.94	0.52
Solyc01g098850.2.1	Short-chain dehydrogenase/reductase family protein	−0.94	0.52
Solyc06g076640.2.1	Tubulin beta chain	−0.94	0.52
Solyc03g116590.2.1	Embryo-specific 3	−0.94	0.52
Solyc06g059740.2.1	Alcohol dehydrogenase 2	−0.93	0.53
Solyc11g042800.1.1	Late embryogenesis abundant protein	−0.93	0.53
Solyc03g025810.2.1	Low-temperature-induced 65 kDa protein	−0.92	0.53
Solyc03g083970.2.1	IQ calmodulin-binding motif family protein	−0.91	0.53
Solyc08g013860.2.1	NAD-dependent malic enzyme 2	−0.90	0.53
Solyc10g078770.1.1	Seed maturation protein LEA 4	−0.90	0.53
Solyc09g090150.2.1	Legumin 11S-globulin	−0.89	0.54
Solyc03g112590.2.1	Cell division protease ftsH homolog	−0.89	0.54
Solyc04g064710.2.1	Alcohol dehydrogenase 2	−0.89	0.54
Solyc00g297330.1.1	Unknown Protein	−0.88	0.54
Solyc07g053360.2.1	Seed biotin-containing protein SBP65	−0.87	0.55
Solyc08g080480.2.1	Unknown Protein	−0.87	0.55
Solyc06g074750.1.1	Histone H2B	−0.86	0.55
Solyc12g098940.1.1	Ubiquitin	−0.85	0.55
Solyc07g032740.2.1	Aspartate aminotransferase	−0.85	0.56
Solyc01g007940.2.1	Alanine aminotransferase 2	−0.83	0.56
Solyc09g065470.2.1	Vicilin	−0.83	0.56
Solyc09g015070.2.1	Reductase 1	−0.83	0.56
Solyc12g014380.1.1	Glucose-6-phosphate isomerase 1	−0.81	0.57
Solyc07g005390.2.1	Aldehyde dehydrogenase	−0.81	0.57
Solyc09g082340.2.1	Vicilin-like protein	−0.80	0.57
Solyc01g107910.2.1	Caffeoyl CoA 3-O-methyltransferase	0.80	1.74
Solyc00g009020.2.1	Mitochondrial ATP synthase	0.80	1.74
Solyc06g063220.2.1	ATP synthase subunit epsilon mitochondrial	0.80	1.74
Solyc02g082090.2.1	Peroxidase	0.80	1.74
Solyc01g102830.2.1	Unknown Protein	0.81	1.75
Solyc00g147570.2.1	Gelsolin	0.81	1.75
Solyc01g080510.2.1	Os05g0406000 protein	0.81	1.75
Solyc08g068220.2.1	50S ribosomal protein L27	0.81	1.75
Solyc03g078000.2.1	High-affinity fructose transporter ght6	0.81	1.76
Solyc03g096840.2.1	Seed specific protein Bn15D1B	0.82	1.76
Solyc06g075810.2.1	NADH dehydrogenase	0.82	1.77
Solyc06g007630.1.1	Ferredoxin	0.82	1.77
Solyc05g007800.2.1	Negatively light-regulated protein	0.83	1.77
Solyc11g072450.1.1	Mitochondrial F0 ATP synthase D chain	0.83	1.78
Solyc10g078450.1.1	U6 snRNA-associated Sm-like protein LSm6	0.83	1.78
Solyc10g011760.2.1	Aldose 1-epimerase family protein	0.84	1.79
Solyc11g066390.1.1	Superoxide dismutase	0.84	1.79
Solyc03g078670.1.1	Unknown Protein	0.85	1.80
Solyc05g053960.2.1	Cysteine-rich extensin-like protein-2	0.85	1.81
Solyc03g097360.2.1	BolA-like	0.85	1.81
Solyc05g056020.2.1	V-type proton ATPase subunit G 2	0.86	1.81
Solyc07g063630.2.1	Vesicle-associated membrane family protein	0.86	1.82
Solyc04g082590.2.1	Canopy homolog 2	0.86	1.82
Solyc02g079750.2.1	Flavoprotein wrbA	0.87	1.82
Solyc02g078540.2.1	Unknown Protein	0.87	1.82
Solyc11g065270.1.1	CHCH domain containing protein	0.87	1.83
Solyc01g007670.2.1	30S ribosomal protein S7 chloroplastic	0.87	1.83
Solyc07g021500.1.1	Unknown Protein	0.87	1.83
Solyc06g083820.2.1	60 ribosomal protein L14	0.88	1.84
Solyc00g072400.2.1	Peroxidase 1	0.88	1.84
Solyc08g006900.2.1	Ribosomal protein L32	0.88	1.84
Solyc10g007350.2.1	Multiprotein bridging factor 1	0.88	1.84
Solyc07g055250.2.1	Cell wall-associated hydrolase	0.89	1.85
Solyc08g075830.2.1	Peroxidase 27	0.89	1.85
Solyc05g041610.1.1	Caffeoyl-CoA O-methyltransferase	0.89	1.86
Solyc07g008350.2.1	Porin/voltage-dependent anion-selective channel protein	0.89	1.86
Solyc11g011340.1.1	Alcohol dehydrogenase	0.90	1.86
Solyc12g094700.1.1	Cathepsin B-like cysteine proteinase	0.90	1.87
Solyc01g049960.2.1	Unknown Protein	0.92	1.89
Solyc03g114970.2.1	Nitrilase associated protein-like	0.92	1.89
Solyc09g082710.2.1	Histone H2A	0.92	1.89
Solyc08g016420.2.1	Prefoldin subunit 6	0.92	1.90
Solyc03g025850.2.1	Remorin 1	0.93	1.91
Solyc01g103220.2.1	Cytochrome c	0.94	1.92
Solyc07g065640.2.1	RPM1 interacting protein 4 transcript 2	0.94	1.92
Solyc05g056290.2.1	Acetyl-CoA carboxylase biotin carboxyl carrier protein	0.95	1.93
Solyc04g049330.2.1	V-type proton ATPase subunit G 1	0.96	1.94
Solyc01g091130.2.1	Nitroreductase	0.96	1.95
Solyc01g095150.2.1	Late embryogenesis abundant protein	0.97	1.96
Solyc07g005240.2.1	FAD-dependent oxidoreductase family protein	0.97	1.97
Solyc01g090360.2.1	Non-specific lipid-transfer protein	0.98	1.97
Solyc01g095050.2.1	Negatively light-regulated protein	0.98	1.98
Solyc04g082010.1.1	Plastocyanin	1.00	1.99
Solyc11g008990.1.1	Phage shock protein A PspA	1.00	2.00
Solyc04g007750.2.1	Major latex-like protein	1.01	2.01
Solyc03g113730.2.1	B12D protein	1.03	2.04
Solyc08g013930.2.1	Peroxidase family protein	1.04	2.05
Solyc01g088140.2.1	Unknown Protein	1.04	2.05
Solyc02g085230.2.1	Nucleolar protein 6	1.04	2.06
Solyc06g036380.1.1	Ulp1 protease family C-terminal catalytic domain containing protein	1.04	2.06
Solyc12g019040.1.1	Exostosin family protein	1.04	2.06
Solyc03g116060.2.1	Gibberellin-regulated protein	1.04	2.06
Solyc06g054520.1.1	3-hydroxyisobutyryl-CoA hydrolase	1.05	2.07
Solyc02g043900.1.1	Unknown Protein	1.06	2.08
Solyc07g041490.1.1	Stress responsive alpha-beta barrel domain protein	1.07	2.09
Solyc04g071580.2.1	Unknown Protein	1.08	2.12
Solyc08g008330.2.1	Unknown Protein	1.09	2.13
Solyc09g074890.1.1	Rapid alkalinization factor 1	1.11	2.15
Solyc04g024840.2.1	GDSL esterase/lipase 1	1.11	2.16
Solyc04g074900.2.1	40S ribosomal protein S21	1.12	2.17
Solyc06g054250.2.1	5&apos-nucleotidase surE	1.14	2.21
Solyc02g092270.2.1	NADH dehydrogenase	1.14	2.21
Solyc12g008950.1.1	At1g17490/F1L3_4	1.15	2.22
Solyc10g076240.1.1	Peroxidase 1	1.21	2.31
Solyc03g113580.1.1	Germin-like protein	1.26	2.40
Solyc03g118110.2.1	Succinate dehydrogenase assembly factor 2, mitochondrial	1.30	2.45
Solyc07g054960.1.1	Myb-related transcription factor	1.35	2.55
Solyc10g005660.2.1	COP9 signalosome subunit 6	1.35	2.55
Solyc11g010160.1.1	Cc-nbs-lrr, resistance protein	1.35	2.55
Solyc06g062770.2.1	At1g17490/F1L3_4	1.36	2.56
Solyc03g117810.2.1	Phosphate import ATP-binding protein pstB 1	1.36	2.57
Solyc11g066270.1.1	Xyloglucan endotransglucosylase/hydrolase 9	1.37	2.59
Solyc05g007090.2.1	Zinc knuckle	1.37	2.59
Solyc01g107990.2.1	MAP protein kinase-like protein	1.42	2.68
Solyc00g015000.1.1	DNA (Cytosine-5-)-methyltransferase 3	1.55	2.93
Solyc02g093230.2.1	Caffeoyl-CoA *O*-methyltransferase	1.55	2.93
Solyc04g028490.1.1	Ulp1 protease family *C*-terminal catalytic domain containing protein	1.55	2.93

^z^ Tomato proteins identified as significantly induced or repressed in tomato radicles from seeds germinated in 50 µM AlK (SO_4_)_2_ in 50 mM Homopipes buffer (pH, 4.5) (treated) and those in the buffer only (control). Tomato seeds were harvested from plants grown in a hydroponic solution supplemented with 50 µM AlK (SO4)_2_. ^y^ Protein accession number is from the ITAG Protein database (release 2.3 on 26 April 2011; Sol Genomics Network, Boyce Thompson Institute, Ithaca, NY, USA). ^X^ protein name annotated in ITAG2.3database. ^w^ The log2 ratio of each protein between treated and control samples measured by the intensity of its constituent peptides. All the listed proteins have passed the *t* test [general linear model (GLM)] with false discovery rate (FDR) corrections (*p* ≤ 0.05), and with a log2 fold change greater than 0.80 (±) which equals to two standard deviations of the near-normal distribution of log2 fold for all proteins identified in the experiment. Statistical analyses were performed using SAS (Version 9.3; SAS Institute Inc. Cary, NC, USA). ^V^ The ratio of protein abundance between treated and control samples, which is antilogarithm of the log2 ratio.
